# Supplementary material for: Evaluation of the Effects of Food Safety Training on the Microbiological Load Present in Equipment, Surfaces, Utensils, and Food Manipulator’s Hands in Restaurants
Source: Microorganisms. 2024 Apr 19;12(4):825. doi: 10.3390/microorganisms12040825 (PMC11052003; doi:10.3390/microorganisms12040825)
Supplement: Supplementary file 1 [file microorganisms-12-00825-s001.zip › microorganisms-2918475-supplementary.pdf]

Section S1: Checklist

I – Physical Facilities and Environment	P	S	N	NA
**Construction and Design (Layout):**<br>• Allows for sanitization (_)<br>• Preservation ( )<br>• Prevents airflow from contaminated areas to clean areas (__)<br>• Layout flow from clean zone to dirty zone (__)<br>• Air quality control and ventilation (__)	5			
**Workspace:**<br>• Physically separated areas (__)<br>• Adequate space for the circulation of people and supporting material (__)<br>• Allows for hygiene operations (__)	3			
**Facilities:**<br>• Clean (__)<br>• Maintained (__)<br>• Well illuminated (__)<br>• Well ventilated (__)<br>• Efficient exhaust system (__)<br>• Suitable room temperature (__)	6			
**Sanitary facilities:**<br>• Separated by gender (__)<br>• Sufficient number (__)<br>• Non-manual flush control (_)<br>• Non-manual faucet control (_)<br>• Faucets with hot and cold water (_)<br>• No direct contact with production areas (_)<br>• Adequate ventilation (_)	7			
**Changing room:**<br>• Separated by gender (__)<br>• Equipped with showers (__)<br>• Washbasins (__)<br>• Clean (__)<br>• Organized (__)<br>• Dimensions adequate for the number of employees (__)<br>• Individual lockers (__)	7			
**Flooring:**<br>• Suitable material (_)<br>• Clean ( )<br>• Well-maintained ( )<br>• Adequate drainage (__)<br>• Non-slip (__)	5			
**Walls:**<br>• Suitable material (_)<br>• Easily washable up to 1.5 m in height ( )<br>• Clean ( )<br>• Well-maintained (__)<br>• Rounded corners (__)	5			

**Ceilings:**<br>• Suitable material (_)<br>• Prevents fire propagation (__)<br>• Clean ( )<br>• Well-maintained ( )<br>• Prevents particle detachment ( )<br>• Lamp protection ( )	6			
**Windows:**<br>• Suitable material (_)<br>• Clean ( )<br>• Well-maintained ( )<br>• Mosquito nets ( )	4			
**Doors:**<br>• Suitable material (__)<br>• Clean (__)<br>• Well-maintained (__)<br>• External access doors closed or equipped with air curtains or screens (__)	4			
**Handwashing facilities:**<br>• In main production areas (_)<br>• Specified washing procedure ( )<br>• Equipped with hot and cold water ( )<br>• Liquid soap ( )<br>• Disinfectant ( )<br>• Adequate drying device (__)	6			
**Water:**<br>• Drinkable at all points ( )<br>• Drinkable for thermal treatment (__)<br>• Non-potable water identified (__)<br>• Treatment of wastewater (__)<br>• Systems or controls to reduce water wastage (__)	5			
**Ice:**<br>• Made from drinkable water (__)<br>• Not contaminated during handling (__)	2			
**Waste or food residue:**<br>• In bins with a lid or plastic bag (__)<br>• Non-manual activation (__)<br>• Clean and well-maintained (__)<br>• Proper and easily cleaned bin (__)<br>• Adequate removal and disposal of food waste (__)<br>• Collection area kept clean and free from animals and pests (__)<br>• Refrigerated area (__)	7			
**Total**	72			

II – Food Handlers	P	S	N	NA
**Clothing/Footwear:**<br>• Ergonomic (__)<br>• Suitable (_)<br>• Complete ( )<br>• Clean ( )<br>• Well-maintained ( )<br>• Exclusively for use in work areas (__)	6			
**Absence of:**<br>• Ornaments (_)<br>• Makeup ( )<br>• Long nails (__)<br>• Nail polish (__)<br>• Dirty nails (__)<br>• Skin lesions ( )<br>• Unprotected lesions (__)<br>• Dirty nails (__)	7			
**Training:**<br>• Adequate training and/or instruction for the correct performance of duties (_)<br>• Constant supervision by a person with appropriate training (__)<br>• Basic training (__)<br>• Periodic training (__)<br>• Additional training (__)	5			
**Health and Occupational Safety:**<br>• Occupational health and safety programs (_)<br>• Handlers in apparent good health (_)<br>• Existence of PPE (Personal Protective Equipment) (__)<br>• Proper use of PPE (__)<br>• PPE used whenever necessary (__)	5			
**Handwashing:**<br>• Frequency (__)<br>• Necessity (__)<br>• For the right amount of time (__)<br>• With suitable product (__)	4			

**Hygienic habits:**<br>• Safe handling behaviour and/or procedure (__)<br>• Correct use of disposable gloves (__)	2			
**Total**	29			

III – Equipment and Utensils	P	S	N	NA
**Surfaces (contact with food):**<br>• Suitable material ( )<br>• Well-maintained ( )<br>• Sanitized ( )	3			
**Equipment:**<br>• Conservation ( )<br>• Maintenance ( )<br>• Not damaged (__)<br>• Cleanliness (__)<br>• Arrangement to facilitate production flow (__)<br>• Automatic control devices (__)<br>• Prevent contamination of food (__)<br>• Exhaust hood where necessary (__)<br>• Good hygiene practices (__)<br>• Allow temperature control and monitoring (__)	10			
**Utensils and Containers:**<br>• Clean (__)<br>• Well-maintained (__)<br>• Suitable material (__)<br>• Separated and identified by area (__)	4			
**Freezing Equipment:**<br>• Sanitization after raw-cooked contact (__)<br>• Frequent sanitization during working shifts (__)<br>• Specific to each task area (__)	3			
**Total**	**20**			

IV – Reception and Storage	P	S	N	NA
**Reception:**<br>• Presence of a responsible person ( )<br>• At the predefined schedule (__)<br>• Reception record (__)<br>• Product verification (__)<br>• Quantity (__)<br>• Temperature (__)<br>• Intact packaging (__)<br>• Transport vehicle (__)<br>• Expiry date (__)<br>• Pre-washing when necessary (__)<br>• Traceability (__)	11			
**Storehouse:**<br>• Ventilation (__)<br>• Absence of humidity (__)<br>• Illumination (__)<br>• Stacking (__)<br>• Distance from the wall (__)<br>• Food placed on pallets and shelves (__)<br>• No crushed or torn packaging (__)<br>• Labels (__)<br>• Expiry dates (__)<br>• FIFO or FEFO (__)<br>• Good conservation status (__)<br>• Cleanliness (__)<br>• Separation and identification of non-compliant products (__)<br>• Grouping by category (__)<br>• Hazardous or non-edible substances labelled and separated (__)<br>• Prevents access to pests (__)<br>• Reduces food deterioration (__)<br>• Absence of food in contact with the floor (__)	18			
**Cold Storage:**<br>• Separate cold equipment for each food ( )<br>• Suitable temperature (__)<br>• Control and recording of temperatures (__)<br>• Temperature change alarm (__)<br>• Foods within the expiration date (__)<br>• Well-packaged foods (__)<br>• With visible labelling (__)<br>• Separation and identification of non-compliant products (__)	8			
**Refrigeration, freezing, and frozen maintenance equipment:**<br>• Conservation (__)<br>• Periodic maintenance (__)<br>• Adequate cleaning (__)<br>• Capacity suitable for the volume of food (__)<br>• With functional temperature verification or recording devices (__)<br>• Electronic or manual recording (__)	8			

• Internal opening system (__)<br>• Adequate lighting (__)				
**Defrosting:**<br>• Done correctly (__)<br>• Defrosting liquids drained (__)<br>• Vegetables go directly for cooking (__)<br>• Defrosting done before cooking whenever necessary (__)<br>• Unused frozen items kept at -18ºC (__)<br>• Defrosted items not refrozen (__)<br>• Food packaging with weight <2.5kg (__)<br>• Defrosted items kept under refrigeration (__)	8			
**Total**	53			

V – Preparation, Cooking, and Pantry	P	S	N	NA
**Raw Foods:**<br>• Well separated from cooked or pre-cooked foods (_)<br>• Handled by different individuals (__)<br>• Raw vegetables and fruits are sanitized (__)<br>• Temperature monitored if necessary (__)	4			
**Frying:**<br>• Fryer with functioning thermostat (_)<br>• Temperature ≤ 180 ºC (_)<br>• Oil filtered before use (___)<br>• Continuous checking of sensory characteristics (_)<br>• Sampling for checking polar compounds (___)<br>• Total oil change if there is oil alteration (_)	6			
**Cooking:**<br>• Ensure safe temperatures (≥ 75ºC) at the core (_)<br>• Use of Pasteurized eggs in preparations that require it (_)<br>• Safe procedures for undercooked meats and eggs (__)	3			
**Post-cooking:**<br>• Short interval until consumption (_)<br>• Unserved foods are immediately refrigerated (__)<br>• Proper maintenance of food temperature (__)<br>• Temperature control (__)	4			
**Dining area:**<br>• Equipment, furniture, and floors sanitized ( )<br>• Replacement in clean containers ( )<br>• Containers with lids ( )<br>• Cold environments at T > 0ºC and <= 5ºC (__)<br>• Hot foods maintained at T >= 65ºC (__)<br>• Temperature controls (__)	6			
**Dining area:**<br>• Equipment, furniture, and floors sanitized (_)<br>• Replacement in clean containers (_)<br>• Containers with lids (_)<br>• Cold environments at T > 0ºC and <= 5ºC (__)<br>• Hot foods maintained at T >= 65ºC (__)<br>• Temperature controls (__)	7			
**Transported foods:**<br>• Closed carts (__)<br>• Temperature > 60ºC for hot foods (__)<br>• Protected in closed containers (__)<br>• Temperature of 4ºC or maximum of 7ºC during transport for cold foods (__)<br>• Frozen foods with temperature < -18ºC with tolerance up to 12ºC during transport (__)	5			
**Regeneration or heating:**<br>• Reaches minimum temperature of 75ºC at the core (__)<br>• Use of suitable equipment for this purpose (__)<br>• Distributed in a short time interval (__)	3			
**Cleaning and Storage Area:**<br>• Suitable facilities (__)<br>• Sanitized (__)<br>• Proper storage of utensils and equipment (__)	7			

• Corrosion-resistant material (__)<br>• Easy to clean (__)<br>• Equipped with hot and cold water (__)<br>• With cleaning equipment and washing machines (__)				
**Washing of delicate dishes:**<br>• Clean area ( )<br>• Well-maintained ( )<br>• Suitable setup ( )<br>• Efficient ( )<br>• Suitable cleaning products (_)<br>• Proper use of products (__)<br>• Hot water under pressure (__)<br>• Proper pre-washing (__)<br>• Mechanical washing (_)<br>• Proper drainage or drying (_)<br>• Proper storage of dishes (_)	11			
**Washing of heavy-duty dishes:**<br>• Clean area ( )<br>• Well-maintained ( )<br>• Suitable setup ( )<br>• Efficient ( )<br>• Suitable cleaning products (_)<br>• Proper use of products (__)<br>• Mechanical washing (_)<br>• Proper drainage or drying (_)<br>• Proper storage of dishes (_)<br>• Presence of 2 sinks in case of manual washing (__)	10			
**Sanitization:**<br>• Suitable products (__)<br>• Proper procedures (__)<br>• Signage (__)<br>• Avoiding contact of chemicals with food (__)	4			
**Total**	69			

VI – Quality Control	P	S	N	NA
**Implementation of procedures and systems:**<br>• Compliance with hygiene requirements (__)<br>• Code of good practices (__)<br>• Code of procedures (__)<br>• Prerequisites of HACCP (__)<br>• Supplier prerequisites and qualification (__)<br>• Specifications document (__)<br>• Control of non-conforming products (__)<br>• Well-displayed various signage (__)	8			
**Traceability:**<br>• Identification and qualification of suppliers (__)<br>• Control of food waste (__)	2			
**Documents and records:**<br>• Occupational health and safety program (__)<br>• Occupational health visits (__)<br>• Training program (__)<br>• Pest control program (__)<br>• Temperatures (__)<br>• Traceability (__)<br>• Non-conformities (__)<br>• Code of good practices (__)<br>• Manufacturing code (__)<br>• Technical data sheets of cleaning products (__)	10			
**Sanitation plan:**<br>• Procedures for washing and disinfection (__)<br>• Definition of areas to be cleaned (__)<br>• Equipment (__)<br>• Suitable materials and procedures (__)<br>• Responsible parties for the processes and areas (__)	5			
**Pest control programs:**<br>• Record (__)<br>• Map (__)<br>• Specific company (__)<br>• Regular rodent and insect control (__)<br>• Absence of pest traces (__)<br>• Insect traps (__)<br>• Window mosquito nets (__)	7			
**Visitors:**<br>• All receive appropriate clothing (__)<br>• They comply with kitchen rules (__)	2			
**Identification of food:**<br>• Labels (__)	8			

• Production date (__)<br>• Type of food (__)<br>• Establishment name (__)<br>• Lot number (__)<br>• Weight (__)<br>• Expiry date (__)<br>• Expiry date after handling (__)				
**Records and microbiological analysis:**<br>• Critical control points (__)<br>• Foods (__)<br>• Surfaces (__)<br>• Handlers (__)<br>• Equipment (__)<br>• Utensils (__)	6			
**Sample:**<br>• Collected daily (__)<br>• All dishes (__)<br>• Stored under refrigeration or freezing (__)<br>• Kept for a minimum of 3 days after consumption of the entire batch (__)<br>• Minimum quantity of 100g (__)<br>• Sterile container (__)<br>• Correct collection procedure (__)<br>• Correct collection time (__)	8			
**Total**	57			

Section S2: Food Safety Questionnaire

Name: _________________________________ Age: _____ Role: __________

True or False:

1- The temperature danger zone for the growth of microorganisms is between 5 to 65ºC. ___

2- Salmonella can only be present in eggs. ___

3- Hand hygiene is one way to prevent food poisoning. ___

4- Freezing food eliminates pathogenic microorganisms. ___

5- Cooked foods cannot be responsible for food poisoning. ___

6- The cooking temperature is sufficient to eliminate all toxins present in the food. ___

7- Bags of carrots and potatoes can be placed directly on the floor. ___

8- Raw fish/meat can be left for more than 30 minutes at room temperature on the countertop? ___

9- Cooked foods should be kept hot at temperatures above 65ºC. ___

10- Burns and wounds can only be covered with bandages. ___

11- Cooked foods should be kept hot at temperatures above 65ºC. ___

12- Waste bins can be left open during service to facilitate waste disposal. ___

13- Is it possible to tell if a surface or food is contaminated just by observation? ___

14- Cooked food and raw food should be handled with different utensils and stored in different places. ___

15- The use of color-coded cutting boards is to differentiate the foods that can be cut on them and prevent cross-contamination? ___

16- All food handlers must maintain a high level of hygiene. ___

17- Should cutting boards be sanded when there are already many cuts or perforations in them? ___

18- Can chemicals be stored next to raw materials? ___

19- Can work uniforms only be worn at the workplace? ___

Multiple Choice:

1. **Of the microorganisms listed below, which ones have you heard of or know of their existence?**

Salmonella	
Clostridium Botulinum	
E. coli	
Yersinia	
Campylobacter jejuni	
Listeria monocytogenes	
Bacillus cereus	
Staphylococcus aureus	

2. **Food poisoning can cause various symptoms. Select the best option:**

a) Vomiting and diarrhoea

b) Fever

c) Nausea and abdominal pain

d) Muscle aches

e) All of the above

3. **The onset of symptoms due to food poisoning takes ____ to appear:**

a) Minutes

b) Hours

c) Days

d) All of the above

Section S3: Qualitative classification in Non-Compliant and Acceptable for *Enterobacteriaceae* and Total Mesophilic Bacteria results.

Enterobacteriaceae

Place of sampling N=208		Restaurant A n	Restaurant B n	Restaurant C n	Restaurant D n
**Equipment**	**NC**	0	0	1	0
**N=56**	**Acc**	12	12	19	12
**Surfaces**	**NC**	8	7	7	2
**N=92**	**Acc**	16	17	14	22
**Utensils**	**NC**	3	3	4	3
**N=60**	**Acc**	13	13	8	13
**Total**		52	52	52	52

Non-compliant (NC) > 1 CFU/cm^2; acceptable (Acc) ≤ 1 CFU/cm^2.

TMB (Total Mesophilic Bacteria)

Place of sampling N=208		Restaurant A n	Restaurant B n	Restaurant C n	Restaurant D n
**Equipment**	**NC**	0	3	4	0
**N=56**	**Acc**	12	9	16	12
**Surfaces**	**NC**	11	9	8	3
**N=92**	**Acc**	13	15	12	19
**Utensils**	**NC**	4	2	5	4
**N=60**	**Acc**	12	14	7	12
**Total**		52	52	52	52

Non-compliant (NC) ≥ 100 CFU/cm²; acceptable (Acc)< 100 CFU/cm².
